# Early consciousness disorder in acute ischemic stroke: incidence, risk factors and outcome

**DOI:** 10.1186/s12883-016-0666-4

**Published:** 2016-08-17

**Authors:** Jie Li, Deren Wang, Wendan Tao, Wei Dong, Jing Zhang, Jie Yang, Ming Liu

**Affiliations:** 1 Department of Neurology, Stroke Clinical Research Unit, West China Hospital, Sichuan University, No. 37, GuoXue Xiang, Chengdu, 610041 Sichuan People’s Republic of China; 2 Department of Neurology, People’s Hospital of Deyang City, Deyang, People’s Republic of China; 3 Department of Neurology, Nanjing First Hospital, Nanjing Medical University, Nanjing, People’s Republic of China

**Keywords:** Ischemic stroke, Early consciousness disorder, Occurrence, Risk factor, Complication, Outcome

## Abstract

**Background:**

Little is known about the incidence and risk factors of early consciousness disorder (ECD) in patients with acute ischemic stroke, or about how ECD may affect complications and outcomes.

**Methods:**

Patients admitted to our hospital within 24 h of onset of acute ischemic stroke were consecutively enrolled. ECD was evaluated clinically and using the Glasgow coma scale. Multivariate analysis was used to identify risk factors of ECD, as well as associations between ECD and clinical outcomes.

**Results:**

Of the 569 patients enrolled, 199 (35 %) had ECD. Independent risk factors of ECD were advanced age (OR 1.027, 95 % CI 1.007 to 1.048), National Institutes of Health Stroke Score on admission (OR 1.331, 95 % CI 1.257 to 1.410), and massive cerebral infarct (OR 3.211, 95 % CI 1.642 to 6.279). ECD was associated with higher frequency of stroke-related complications (83.4 % vs. 31.1 %, *P* < 0.001) and higher in-hospital mortality (17.1 % vs. 0.5 %, *P* < 0.001). ECD independently predicted 3-month death/disability (OR 3.272, 95 % CI 1.670 to 6.413).

**Conclusions:**

ECD is prevalent in Chinese patients with acute ischemic stroke. Risk factors include advanced age, stroke severity, and massive cerebral infarct. ECD is associated with higher frequency of stroke-related complications and 3-month death/disability.

## Background

Stroke is the second most frequent cause of death and the most frequent cause of disability worldwide. In China, stroke has become the leading cause of death and disability in both urban and rural populations, with incidence increasing every year [1]. Approximately 80 % of all strokes are ischemic [1].

Consciousness, which refers to awareness of self and environment, depends on levels of arousal and wakefulness, as well as stimulus content [2]. Many patients in early stages of acute ischemic stroke show acute disorder of consciousness, a condition known as early consciousness disorder (ECD). In fact, stroke is one of the three most frequent causes of conscious disturbance in emergency rooms, together with trauma and hypoglycemia [3]. Data from various stroke registries suggest that 4–38 % of stroke patients experience decreased level of consciousness or coma, and 13–48 % experience confusion or delirium [4–11].

Little is known about the incidence or risk factors of ECD in patients who experience acute ischemic stroke. This is an important question, because early consciousness disorder may disturb physician’ history-taking in clinical practice and ECD in the acute stroke period may predict stroke progression and increase risk of stroke-associated pneumonia [12–14]. Studies have found several possible risk factors of acute disorder of consciousness after stroke: age, sex, previous stroke, atrial fibrillation, diabetes mellitus, alcohol consumption, stroke severity, infarct sites and, according to some reports, massive cerebral infarct and multiple brain infarcts [15–19]. However, currently available information are rough data extracted from registries for different population. Direct comparative data between stroke patients with ECD and without ECD especially on prospective study are scarce. Further work is needed to identify a reliable set of ECD predictors.

Also unclear is whether ECD influences stroke-related complications and clinical outcomes in stroke patients. This is made more important by the fact that the presence of ECD in a stroke patient can affect decisions about treatment and management.

To address these questions, the present prospective study analyzed potential ECD risk factors and associated outcomes in a mid-sized cohort of Chinese patients with acute ischemic stroke.

## Methods

### Subjects

Between 1 January 2009 and 31 December 2010, patients with first-ever or recurrent stroke who were admitted to the Department of Neurology at West China Hospital of Sichuan University were consecutively and prospectively registered into the Chengdu Stroke Registry database as described [20]. We enrolled into the present study only those patients who were admitted within 24 h of symptom onset and whose diagnosis of ischemic stroke was confirmed by computed tomography (CT) or magnetic resonance imaging (MRI) of the brain. We excluded patients who had previously received a modified Rankin Scale (mRS) score >2 or who had premorbid conditions such as trauma, intoxication, infection, metabolic or other systemic disease.

### Baseline assessment

Using a standardized form, we collected patient data at baseline that included age, sex, time between stroke onset and admission, initial stroke severity [assessed using the National Institutes of Health Stroke Scale (NIHSS) score], systolic and diastolic blood pressure, serum glucose concentration, Glasgow coma scale (GCS) score, stroke risk factors and results of neurological imaging. Stroke risk factors in the present study included hypertension, diabetes mellitus, hyperlipidemia, coronary heart disease, atrial fibrillation, valvular heart disease, previous stroke, history of head trauma, current smoking and alcohol consumption [20]. Neurological imaging findings analyzed in this study were where infarcts occurred (frontal, parietal, temporal, occipital and insular lobe, basal ganglia, subcortical white matter, thalamus, brain stem, cerebellum), whether the left or right hemisphere was involved, and whether the posterior or anterior circulation was involved [21, 22].

Patients in which CT or MRI revealed involvement of more than half the middle cerebral artery distribution were diagnosed as having massive cerebral infarct [23]. Multiple brain infarction was defined as multiple recent infarcts involving non-contiguous regions of abnormality across multiple vascular territories [24]. TOAST criteria were used to assign the likely stroke etiology in each patient as large-artery atherosclerosis, cardioembolism, small-artery occlusion, other determined etiology or undetermined etiology [25].

Conscious state at baseline (initially when admitted to the neurology ward) was independently assessed by two experienced neurologists according to Adams and Victor’s Principles of Neurology (9th edition) [26], via evaluating the patient’s wakefulness, verbal and motor response, orientation to person, place and time, and other physical examination. Patients classified as impaired consciousness (Somnolence, Stupor, Coma, Confusion or Delirium) were assigned to the ECD group, while others were assigned to the no-ECD group.

### In-hospital and post-discharge outcomes

Hospital staff determined in-hospital stroke-related complications and mortality based on record review after patient discharge or death. At 3 months after stroke onset, patients were contacted for follow-up and asked to fill out a questionnaire during a structured telephone interview or by post. The primary outcomes in this study were in-hospital death and either death or disability during the 3-month follow-up. Disability was defined as an mRS score of 3–5 [27].

### Statistical analysis

All statistical analyses were performed using SPSS for Windows 16.0 (IBM, Chicago, IL, USA). Inter-group differences for continuous variables were assessed for significance using Student’s *t*-test or the Mann-Whitney *U* test, while differences for categorical variables were assessed using the chi-squared and Fisher exact tests. Multivariate logistic regression by a backward stepwise procedure was used to identify independent risk factors of ECD on admission. Variables were eliminated from the model if their associated P was >0.10. Logistic regression was used to identify independent predictors of 3-month death/disability. When appropriate, effect sizes were estimated using odds ratios (ORs) and 95 % confidence intervals (95 % CIs). All significance levels mentioned are 2-tailed, and the significance threshold was defined as *P* < 0.05.

## Results

Between 1 January 2009 and 31 December 2010, 1,506 patients with acute ischemic stroke were consecutively and prospectively registered in the Chengdu Stroke Registry. Of those, 569 (37.8 %) were admitted to our hospital within 24 h of stroke onset and were therefore included in the present study. Of this group, comprising 303 men and 266 women aged 64.75 ± 14.02 years, 199 (35 %) had ECD on admission, of whom 38 (6.7 %) were Coma. Among the 143 patients in our cohort with hyper-acute ischemic stroke who were admitted within 6 h of symptom onset, ECD occurred in 52 (36.4 %). Among the 81 patients admitted within 4.5 h of symptom onset, ECD occurred in 29 (35.8 %).

### ECD and baseline characteristics

Comparison of baseline characteristics of patients with or without ECD (Table 1) showed that those with ECD were older, had higher serum glucose concentrations, higher median NIHSS score and lower median GCS score. Patients with ECD also showed higher rates of several stroke risk factors (coronary heart disease, atrial fibrillation and valvular heart disease), but a lower rate of hyperlipidemia.

**Table 1:** Baseline characteristics of Chinese patients with acute ischemic stroke and with or without early consciousness disorder

	With ECD	Without ECD	*P*
Patients, n (%)	199 (35.0)	370 (65.0)	——
Mean age, yr	66.7 ± 13.6	63.7 ± 14.2	0.016*
Women, n (%)	103 (51.8)	163 (44.1)	0.079***
Delay from symptom onset to admission, h	13.9 ± 8.2	15.0 ± 8.5	0.130*
Median NIHSS score on admission	16 (0–33)	3.5 (0–19)	<0.001**
Median GCS score on admission	10 (3–15)	15 (9–15)	<0.001**
SBP ≥180 mmHg, n (%)	16 (8.0)	24 (6.5)	0.489***
DBP ≥ 100 mmHg, n (%)	32 (16.1)	43 (11.6)	0.134***
Serum glucose on admission, mmol/L	8.53 ± 3.62	7.21 ± 2.85	<0.001*
Stroke risk factors, n (%)			
Hypertension	119 (59.8)	237 (64.1)	0.317***
Diabetes mellitus	56 (28.1)	90 (24.3)	0.320***
Hyperlipidemia	34 (17.1)	97 (26.2)	0.014***
Coronary heart disease	29 (14.6)	28 (7.6)	0.008***
Atrial fibrillation	85 (42.7)	70 (18.9)	<0.001***
Valvular heart disease	39 (19.6)	35 (9.5)	0.001***
Current smoking	43 (21.6)	101 (27.3)	0.137***
Alcohol consumption	38 (19.1)	57 (15.4)	0.260***
Previous stroke	34 (17.1)	77 (20.8)	0.285***
History of head trauma	2 (1.0)	5 (1.4)	1.000***

*Abbreviations: DBP* diastolic blood pressure, *ECD* early consciousness disorder, *GCS* Glasgow coma scale, *NIHSS* National Institutes of Health Stroke Scale, *SBP* systolic blood pressure

*Student’s *t* test

**Mann–Whitney *U* test

***χ ^2^ test

All patients in the cohort underwent neuro-imaging by CT (483, 84.9 %), MRI (489, 85.9 %) or both (405, 71.2 %). Comparison of imaging findings between patients with or without ECD (Table 2) showed that those with ECD had significantly higher rates of cerebral cortex infarction, midbrain infarction, massive cerebral infarction and anterior circulation involvement.

**Table 2:** Neurological imaging findings in Chinese patients with acute ischemic stroke and with or without early consciousness disorder

	With ECD	Without ECD	*P*
Infarct site, n (%)			
Frontal lobe	115 (57.8)	79 (21.4)	<0.001
Parietal lobe	119 (59.8)	90 (24.3)	<0.001
Temporal lobe	133 (66.8)	89 (24.1)	<0.001
Occipital lobe	56 (28.1)	37 (10.0)	<0.001
Insula lobe	22 (11.1)	22 (5.9)	0.030
Subcortical white matter	35 (17.6)	100 (27.0)	0.012
Basal ganglia	92 (46.2)	184 (49.7)	0.408
Thalamus	15 (7.5)	51 (13.8)	0.026
Cerebellum	22 (11.1)	24 (6.5)	0.057
Midbrain	11 (5.5)	7 (1.9)	0.018
Pons	16 (8.0)	47 (12.7)	0.091
Medulla	2 (1.0)	13 (3.5)	0.075
Massive cerebral infarction, n (%)	124 (62.3)	37 (10.0)	<0.001
Multiple brain infarction, n (%)	61 (30.7)	136 (36.8)	0.144
AC involved, n (%)	172 (86.4)	281 (75.9)	0.003
PC involved, n (%)	43 (21.6)	131 (35.4)	0.001
Left hemisphere involved, n (%)	109 (54.8)	196 (53.0)	0.681
Right hemisphere involved, n (%)	103 (51.8)	188 (50.8)	0.829

*Abbreviations: AC* anterior circulation, *PC* posterior circulation

*χ* ^2^ test

Patients with ECD showed lower rates of subcortical white matter infarction, thalamus infarction and posterior circulation involvement. The two patient groups did not differ significantly in rates of multiple brain infarction or involvement of the left or right hemispheres.

The distribution of four conscious states differed significantly among the TOAST stroke subtypes (*P* < 0.001). The subtype with the highest incidence of ECD was cardioembolism (56.1 %), followed by stroke of other determined etiology (52.3 %), large-artery atherosclerosis (39.6 %), stroke of undetermined etiology (33.0 %), and small-artery occlusion (11.0 %).

### Risk factors of ECD on admission

Multivariable logistic regression identified several independent risk factors of ECD in acute ischemic stroke patients admitted within 24 h of stroke onset (Table 3): age (adjusted OR 1.027, 95 % CI 1.007 to 1.048), NIHSS score on admission (adjusted OR 1.331, 95 % CI 1.257 to 1.410), massive cerebral infarction (adjusted OR 3.211, 95 % CI 1.642 to 6.279), high serum glucose on admission (adjusted OR 1.141, 95 % CI 1.055 to 1.235), and history of alcohol consumption (adjusted OR 2.123, 95 % CI 1.030 to 4.375). Conversely, high systolic pressure on admission appeared to protect against ECD within 24 h of stroke onset (adjusted OR 0.983, 95 % CI 0.968 to 0.999). Anterior circulation involvement did not independently predict ECD (adjusted OR 1.17, 95 % CI 0.560 to 2.441, *P* = 0.677).

**Table 3:** Multivariate logistic regression to identify risk factors for early consciousness disorder on admission in Chinese patients with acute ischemic stroke

Variable	Adjusted OR^a^ (95 % CI)
Age	1.027 (1.007 to 1.048)
NIHSS on admission	1.331 (1.257 to 1.410)
Serum glucose on admission	1.141 (1.055 to 1.235)
Massive cerebral infarction	3.211 (1.642 to 6.279)
History of alcohol consumption	2.123 (1.030 to 4.375)
High systolic pressure	0.983 (0.968 to 0.999)

^a^Adjusted for age, sex, stroke risk factors, stroke severity and imaging findings

### Stroke-related complications

The rate of stroke-related complications (excluding stroke recurrence) was higher among patients with ECD than among patients without ECD (83.4 % vs. 31.1 %, *P* < 0.001; Table 4). The most frequent neurological complication in patients with ECD was malignant edema (30.2 %), followed by hemorrhagic transformation (22.1 %) and post-stroke epilepsy (11.1 %). Pulmonary infection was the most frequent medical complication among patients with ECD (58.3 %).

**Table 4:** In-hospital, stroke-related complications in Chinese patients with acute ischemic stroke and with or without early consciousness disorder

	With ECD	Without ECD	*P*
All complications, n (%)	166 (83.4)	115 (31.1)	<0.001
Neurological complications, n (%)			
Brain oedema	60 (30.2)	6 (1.6)	<0.001
Haemorrhagic transformation	44 (22.1)	26 (7.0)	<0.001
Seizures and epilepsy	22 (11.1)	6 (0.6)	<0.001
Central hyperthermia	7 (3.5)	1 (0.3)	0.006
Recurrent stroke	3 (1.5)	3 (0.8)	0.730
Medical complications, n (%)			
Pulmonary infection	116 (58.3)	64 (17.3)	<0.001
Urinary tract infection	18 (9.0)	10 (2.7)	0.001
Gastrointestinal bleeding	34 (17.1)	17 (4.6)	<0.001
Electrolyte disturbance	81 (40.7)	49 (13.2)	<0.001
Acute renal failure	17 (8.5)	6 (1.6)	<0.001
Urinary incontinence	49 (24.6)	12 (3.2)	<0.001
Bed sores	10 (5.0)	3 (0.8)	0.004
Phlebothrombosis	6 (3.0)	1 (0.3)	0.015
Falls	5 (2.5)	0 (0)	0.010

*χ* ^2^ test

### Outcomes

Of the 569 patients in our cohort, 29 (5.1 %) were lost during 3-month follow-up, of whom 13 had ECD on admission and 16 did not (*P* = 0.253). Of the remaining 540 patients, those with ECD showed significantly higher rates of in-hospital mortality (17.1 % vs. 0.5 %, *P* < 0.001), death during 3-month follow-up (33.9 % vs. 3.1 %, *P* < 0.001) and death/disability (mRS 3–5) during follow-up (78.0 % vs. 21.8 %, *P* < 0.001). Multivariate logistic regression in which data were adjusted for age, sex and stroke risk factors identified age, GCS score and ECD as independent predictors of death and death/disability at 3 months (Table 5). When NIHSS score and stroke-related complications were included as inputs in this multivariate model, ECD was still the independent predictor of death/disability at 3 months (adjusted OR 3.272, 95 % CI 1.670 to 6.413), but was not the independent predictor of death at 3 months (adjusted OR 1.641, 95 % CI 0.749 to 3.592).

**Table 5:** In-hospital and 3-month outcomes of Chinese patients with acute ischemic stroke and with or without early consciousness disorder

	With ECD	Without ECD	OR (95 % CI)	*P*	Adjusted OR^a^ (95 % CI)
In-hospital death, n (%)	34 (17.1)	2 (0.5)	37.9 (9.0 to 159.6)	<0.001	3.2 (0.5 to 19.5)
Loss to 3-month follow-up, n (%)	13 (6.5)	16 (4.3)	——	0.253	——
3-month deaths, n (%)	63 (33.9)	11 (3.1)	13.2 (7.0 to 25.1)	<0.001	1.6 (0.8 to 3.6)
3-month death/disability	145 (78.0)	77 (21.8)	12.7 (8.3 to 19.5)	0.001	3.3 (1.7 to 6.4)

^a^Adjusted for age, sex, stroke risk factors, stroke severity and stroke-related complications

## Discussion

Although a sizeable proportion of stroke patients are admitted to the neurology ward with conscious disturbance, the incidence and risk factors of ECD are poorly understood, as are potential relationships between ECD and clinical outcomes in acute ischemic stroke. Here we provide evidence that ECD is common in Chinese patients with acute ischemic stroke, occurring in more than one of every three patients in our cohort that was admitted within 24 h of symptom onset. In this subset of patients who had ECD, nearly a third was in a state of decreased consciousness or coma on admission. This incidence is within the range of 4.3–37.7 % reported by other stroke registries [4–9]. In contrast, the 5.3 % incidence of delirium in our study is lower than the range of 13–48 % reported for other ethnic groups [10, 11]. Differences in the incidence of consciousness disorder between our study and others may easily arise from heterogeneity in the time between stroke onset and hospital admission, which most studies in the literature did not strictly control. Such heterogeneity increases the risk that pre-hospital care, disease course and stroke-related complications contributed to observed conscious disturbance. Another simple explanation for differences between our study and others is that ours involved a single hospital, highlighting the fact that our findings should be validated in multi-site populations of Asian and non-Asian ethnicities.

Incidence of ECD was highest among the subgroup of patients with cardioembolism, and lowest among the subgroup with small-artery occlusion. These results are consistent with a study showing that altered consciousness was 3.2-fold more likely to occur in patients with cardioembolic infarction than in patients with atherothrombotic infarction [28]. In addition, the European Community Stroke Project reported that the incidence of coma and confusion within a week of onset of acute ischemic stroke was higher in the presence of atrial fibrillation than in its absence [17]. This may be because cardiac emboli are often large and therefore more likely to affect the main arteries of the cerebral vascular system, which then causes severe stroke [29].

In our cohort, age, NIHSS score on admission, massive cerebral infarction, high serum glucose on admission and history of alcohol consumption were independent risk factors of ECD. These results are consistent with a cross-sectional study of 9,044 Caucasian prehospital patients that showed that patients with abnormal GCS scores were more likely than patients with normal scores to be male and slightly older and to have a history of alcohol use, diabetes, substance abuse, stroke/transient ischemic attack and seizure [19]. Contrary to studies in French cohorts [18], our results do not support the idea that involvement of anterior circulation and multiple infarctions independently influence early conscious state in patients with acute ischemic stroke. In our cohort, CT/MRI imaging confirmed massive cerebral infarction in nearly two-thirds of patients with ECD, and 20 % of these patients suffered from hemorrhagic transformation during hospitalization. And for this reason, ECD should be considered an important sign in patients with acute ischemic stroke, and it should be taken into account when designing treatment and management strategies.

Our results suggest that poor glycemic control is a predictor of ECD in diabetic patients with acute ischemic stroke. History of diabetes mellitus was identified as an independent predictor of ECD in patients with acute ischemic stroke only when the multivariate logistic regression model did not include serum glucose concentration on admission. This result may reflect the fact that hyperglycemia increases coagulation and inhibits fibrinolysis, decreasing reperfusion of ischemic tissue and increasing infarct volumes. Hyperglycemia inhibits vasodilation, increasing the production of reactive oxygen species and nicotinamide adenine dinucleotide phosphate. Hyperglycemia may also inhibit mitochondrial function in the ischemic penumbra, causing substantial intracellular acidosis [30]. Previous work has associated hyperglycemia with a higher rate of hemorrhagic complications in patients with acute ischemic stroke [30]. Our results, together with previous work, suggest that close monitoring and control of serum glucose in diabetic patients with ischemic stroke may reduce ECD incidence and improve prognosis. However, as patients in our cohort were not given hemoglobin A1c (HbA1c) detection routinely, the interpretation of our results should be cautious because we couldn’t rule out the interference from stress hyperglycemia as a component of the acute phase response caused by severe stroke.

Interestingly, we found evidence that high systolic pressure on admission protects against ECD in patients with acute ischemic stroke. This may reflect auto-regulation of cerebral blood flow during the acute stage of infarction. Indeed, an earlier prospective study from our group showed that a decrease in blood pressure during the first 24 h after admission independently predicts one-month death in Chinese patients with acute stroke [31]. A prospective study in Spanish patients with acute ischemic stroke found that both higher and lower blood pressure during the first 24 h after stroke onset are associated with early neurological deterioration and poor prognosis [32]. Together, these studies highlight the importance of closely monitoring and controlling blood pressure during the first 24 h after stroke. In particular, they highlight the need to prevent a reduction in blood pressure in early stages of infarction. A study of 22 older American patients with delirium using 99mTc HMPAO SPECT scanning associated this conscious state with reduced blood flow in the left inferior frontal, right temporal, right occipital, and pontine regions [33]. It may be that a decrease in systemic blood pressure reduces cerebral perfusion pressure and blood flow to the ischemic penumbra, particularly in the absence of normal auto-regulation [34]. Preventing a reduction in blood pressure soon after infarction is consistent with official guidelines for early management of acute ischemic stroke [35].

Post-stroke complications, both neurological and medical, account for 23–50 % of deaths related to acute ischemic stroke. In particular, brain edema is a major cause of death during the first week after stroke [15]. Medical and neurological complications can hinder rehabilitation, prolong hospitalization, increase healthcare costs and contribute to poor functional outcomes or death [36]. The present study associates ECD with significantly elevated risk of stroke-related complications. The most frequent neurological complication in our patients with ECD was malignant edema, followed by hemorrhagic transformation and post-stroke epilepsy. Regarding post-stroke seizures or epilepsy, the overall frequency is similar to previous reports [15]. As seizures or epilepsy is not the primary issue of the present study, patients in our cohort were not given electroencephalography routinely and we didn’t record detailed information about acute symptomatic seizures which could result in early consciousness disorder. Pulmonary infection was the most common medical complication. Our results are consistent with work in US stroke patients associating greater neurological deficit with higher incidence of medical complications; indeed, that study found neurological impairment to be the strongest predictor of medical complications [37]. Since NIHSS score on admission was significantly higher among our patients with ECD than among patients without it, we speculate that more severe stroke at least partly explains the higher frequency of stroke-related complications in stroke patients with ECD.

Many complications are preventable or treatable if recognized in time. Therefore clinicians should pay close attention to patients with acute ischemic stroke and ECD in order to detect stroke-related complications as soon as possible, and thereby improve stroke outcome. Our results, and those of previous studies, highlight the need for large trials addressing the prevention and treatment of complications and their effect on stroke outcome in this stroke patient subpopulation. Indeed, the present study is one of the few to directly compare characteristics and prognosis of acute ischemic stroke patients with and without ECD. Our results suggest that the two populations differ in several clinically important respects, justifying large, well-designed studies of early management of stroke patients with ECD.

In our cohort, ECD independently predicted death during hospitalization and 3-month follow-up according to a multivariate regression model that did not take into account NIHSS score on admission or stroke-related complications. When these two parameters were included as inputs in the model, ECD was no longer an independent predictor of these outcomes, although it remained an independent predictor of 3-month death/disability. These different results echo discrepancies between previous studies showing associations of ECD with post-stroke death and poor functional outcome [38] or no such associations [39]. While these discrepancies may reflect differences in hospital populations and enrolment criteria, they are consistent with the idea that neurological deficit and stroke-related complications confound the association of ECD with death. The present study, to our knowledge, provides the first evidence that ECD elevates (3.3-fold) the risk of 3-month death/disability in patients with acute ischemic stroke, even after adjusting for confounding factors. This result is consistent with a study in French patients with acute stroke that associated acute confusional state with worse 6-month functional outcome [40].

The results of the present study should be interpreted with caution given its limitations. First, the cohort came from a single hospital, so the results may not be representative of other populations. Second, we conducted follow-up at a single time point of only 3 months, and follow-up involved a telephone interview or mailed questionnaire instead of a clinical visit, which may increase the risk of reporting bias.

## Conclusions

The present study provides clear evidence that at least in Chinese populations, ECD may occur in as many as one-third of patients with acute ischemic stroke, particularly those with cardio-embolism stroke. Our analysis identifies age, stroke severity, and massive cerebral infarction as potential risk factors of ECD in ischemic stroke patients. Although ECD appears to affect anterior circulation much more often than posterior circulation, involvement of anterior circulation does not appear to independently predict ECD in patients with acute ischemic stroke. ECD in such patients is associated with malignant edema, hemorrhagic transformation and epilepsy, as well as with death and disability within 3 months after stroke onset.

## Abbreviations

ECD: Early consciousness disorder
CT: Computed tomography
MRI: Magnetic resonance imaging
mRS: Modified Rankin Scale
NIHSS: National Institutes of Health Stroke Scale
GCS: Glasgow coma scale
ORs: Odds ratios
95 % CIs: 95 % confidence intervals
